# Topics of Public Interest

**Published:** 1904-08

**Authors:** 


### TOPICS OF PUBLIC INTEREST.

### Examination for Army Medical Service.

THE examination of applicants for commission in the medical corps of the army will be materially modified after July 1, 1904, when the amended regulations governing the matter will go into effect. Immediate appointment of applicants after successful physical and professional examination,—the latter embracing all subjects of a medical education,—will be discontinued; thereafter, applicants will be subjected to a preliminary examination and a final or qualifying examination with a course of instruction at the army medical school in Washington intervening.

The preliminary examination will consist of a rigid inquiry into the physical qualifications of applicants and written examination in the following subjects: mathematics (arithmetic, algebra and plane geometry); geography; history (especially of the United States); Latin grammar and reading of easy Latin prose; English grammar, orthography, composition; anatomy; physiology; chemistry and physics; materia medica and therapeutics; normal histology. The subjects in general education above mentioned are an essential part of the examination and cannot under any circumstances be waived.

The preliminary examination will be conducted concurrently throughout the United States by boards of medical officers at most convenient points; the questions submitted to all applicants will be identical thus assuring a thoroughly competitive feature; and all papers will be criticised and graded by an army medical board in Washington. Applicants who attain a general average of 80 per cent. and upwards in this examination will be employed as contract surgeons and ordered to the army medical school for instruction as candidates for admission to the medical corps of the army; if, however, a greater number of applicants attain the required average than can be accommodated at the school, the requisite number will be selected according to relative standing in the examination.

The course of instruction at the army medical school will consist of lectures and practical work in subjects peculiarly appropriate to the duties which a medical officer is called upon to perform. While at this school the students will be held under military discipline, and character, habits, and general deportment closely observed.

The final or qualifying examination will be held at the close of the school term and will comprise the subjects taught in the school, together with the following professional subjects not included in the preliminary examination: surgery; practice of medicine; diseases of women and children; obstetrics; hygiene; bacteriology and pathology; general aptitude will be marked from observation during the school term. A general average of 80 per cent. in this examination will be required as qualifying for appointment, and candidates attaining the highest percentages will be selected for commission to the extent of the existing vacancies in the medical department. Candidates who attain the requisite general average who fail to receive commissions will be given certificates of graduation at the school and will be preferred for appointment as medical officers of volunteers or for employment as contract surgeons; they will also be given opportunity to take the qualifying examination with the next succeeding class.

It is not thought that, for the present at least, the number successfully passing the preliminary examination will be greater than can be accommodated at the army medical school, nor that the number qualifying for appointment will exceed the number of vacancies. If, however, the class of candidates qualifying should be larger than reasonably thought, the young physicians who fail to receive commissions will not have wasted their time, as the course of instruction at the school, while in a large measure specialised to army needs, is such as will better fit them for other professional pursuits, and furthermore they will have received a fair compensation while under instruction.

Admission to the preliminary examination can be had only upon invitation from the surgeon-general of the army, issued after formal application to the secretary of war for permission to appear for examination. No applicant, whose age exceeds thirty years, will be permitted to take the examination, and the authorities at the war department desire it distinctly understood that this limit of age will be rigidly adhered to. Hospital training and practical experience are essential requisites, and an applicant will be expected to present evidence of one year's hospital experience or its equivalent (two years) in practice.

The first preliminary examination under the amended regulations above referred to will be held about August 1, 1904; those desiring to enter the same should at once communicate with the surgeon-general of the army, Washington, D. C., who will be pleased to furnish all possible information in regard thereto.

---

### Substitution Has Again Been Punished.

SUPREME COURT, STATE OF NEW YORK,
COUNTY OF NEW YORK.

FAIRCHILD BROTHERS AND FOSTER, a Corporation,

Plaintiff, against

MORRIS DLUGASCH and HERMAN FINKELSTEIN, doing business under the firm name or style of Broadway Drug Company,

Defendants.

The summons in this action, dated the 20th day of October, 1903, and the complaint herein, verified the 20th day of October, 1903, having been duly served on the defendants on the 21st day of October, 1903, together with an order to show cause, containing a preliminary injunction against the defendants and each of them, dated the 21st day of October, 1903, and an undertaking having been filed by the plaintiff herein and duly approved by the court, and an order of injunction pendente lite having been granted and entered herein on the 30th day of November, 1903; and the defendants having answered by their answer verified the 9th day of November, 1903, and having on the 23d day of March, 1904, offered in writing to allow judgment to be taken against them to the effect that "the said defendants and each of them, and their servants, agents and employes, and all persons acting in their behalf, be prohibited, restrained and enjoined perpetually from selling, dispensing, advertising or displaying at the drug store of said defendants at No. 177 Broadway, Borough of Manhattan, City of New York, or elsewhere, any chemical or pharmaceutical preparations of any sort or kind whatsoever bearing signs, labels or wrappers marked 'Fairchild' or 'Dr. Fairchild,' or any similar word or words, or purporting to be made by 'Dr. Fairchild' or 'Fairchild,' which said preparations are not manufactured by plaintiff;" and the plaintiff, on the 23d day of March, 1904, the same being within ten (10) days after service of said offer of judgment having accepted said offer, as appears by the affidavit of Arthur F. Gotthold, duly verified the 23d day of March, 1904, and hereto annexed; and the parties herein having adjusted the money damages and costs as prayed for in the complaint;

Now on motion of Gould & Wilkie, attorneys for the plaintiff herein, it is

ADJUDGED that the defendants and each of them and their servants, agents and employes, and all persons acting in their behalf, be and they hereby are prohibited, restrained and enjoined perpetually from selling, dispensing, advertising or displaying at the drug store of said defendants at No. 177 Broadway, Borough of Manhattan, City of New York, or elsewhere, any chemical or pharmaceutical preparations of any sort or kind whatsoever bearing signs, labels or wrappers marked "Fairchild" or "Dr. Fairchild," or any similar word or words, or purporting to be made by "Dr. Fairchild" or "Fairchild," which said preparations are not manufactured by plaintiff.

THOS. L. HAMILTON, *Clerk.*

Dated, March 24, 1904.

---

### A SUBSTITUTE FOR RUBBER GLOVES.

J. B. MURPHY is now experimenting with a temporary covering for the hands while operating. He immerses his hands in a 4 to 8 per cent. solution of gutta percha in benzine. This leaves the hands covered by an impervious and sufficiently durable rubber coating. This film is very thin and does not interfere with the delicacy of touch. The false skin can readily be removed by washing the hands in benzine.

